# Clinical and Social Outcomes five years after closing a mental hospital: a trial of cognitive behavioural interventions

**DOI:** 10.1186/1745-0179-1-25

**Published:** 2005-11-23

**Authors:** Antonino Mastroeni, Carla Bellotti, Esterina Pellegrini, Francesco Galletti, Elena Lai, Ian RH Falloon

**Affiliations:** 1 Department of Mental Health, Appiano Gentile, Como, Itlay; 2 Department of Psychiatry, University of Auckland, New Zealand and ARIETE, 06050 Mercatello (PG), Italy

**Keywords:** Mental Hospital Closure, Evidence-Based Treatment, Controlled trial

## Abstract

**Background:**

To investigate the outcome of patients transferred from hospital to community care in Como, Italy after 6 months intensive psychosocial rehabilitation prior to discharge.

**Method:**

All 149 residents with a primary psychiatric diagnosis were assigned to receive either a 6-month pre-discharge course of goal-oriented rehabilitation, (IT), or routine management, (RT). BPRS and GAF ratings were made by blind, independent assessors before and at 12, 24, 36, 48, and 60 months after discharge and the results examined with repeated measures analysis of variance.

**Results:**

Overall change in residence was achieved without any major detriment to the health and welfare of most patients. The cohort of patients who received intensive rehabilitation, (IT), prior to discharge showed significantly lower impairment and disability throughout the five years compared to the cohort receiving routine management, (RT), prior to discharge. Total BPRS scores remained significant when initial differences in the cohorts were covaried, whereas GAF failed to remain significant (p = 0.051).

**Conclusion:**

The treatment provided prior to transfer from long-stay hospital to community residence may have long-term clinical benefits for chronically disabled patients.

## Introduction

Despite overwhelming evidence that institutional care is ineffective and often harmful for chronically impaired psychiatric patients, most policies to close long-term mental hospitals have been politically rather than professionally driven. Nowhere has that been more evident than in Italy. In 1978 the Italian Law 180 prevented the admission of any new cases to long-stay hospitals. This was followed in 1994 and 1995 by further national and local laws that aimed to accelerate the closure of mental hospitals that had been progressing very slowly [1]. The most recent laws fined Local Health Units and Hospitals if they did not close their mental hospitals and relocate patients to community housing before the end of 1999. One-third of the residents of these dilapidated hospitals were mentally ill, while another third were elderly or demented, and the remainder intellectually handicapped. Thus, the program to relocate patients to community housing was complex.

The benefits of community living for long-term severely disabled mentally disordered people have been fiercely debated [2-4]. A controversial problem has been the management of those behaviourally disturbed patients who are prone to violence or sexual misbehaviour who have been rehoused without the 24-hour supervision provided in hospitals [5]. Few prospective surveys have documented the process of closing mental hospitals and much of the debate has centred on media presentations associated with rare incidents of criminal violence or problems of homelessness. The most comprehensive study, the Friern Hospital Project, was carried out in London in the 1980s [2]. Extensive documentation provided a benchmark for patient relocation programmes of this kind. Other studies have provided less detailed reports of similar projects [6-8].

Although rehousing is the essential component of these hospital closure projects, the manner in which patients are prepared for this stressful lifestyle change should not be underestimated. Transitions of this kind offer an opportunity to review treatment and to ensure that evidence-based methods are applied for residual clinical and social morbidity, as well as to prevent exacerbations during the life change process and beyond.

We are aware of only one controlled trial of treatment methods used in the transition of long-term hospital patients to community care [9]. This study demonstrated that a structured educative programme using basic cognitive behavioural methods was more successful in achieving successful community tenure than milieu therapy or traditional supportive care.

The Como Project aimed to address both patient and professional competence for life and work in community settings. The key variable was training all staff in current evidence-based goal- and problem-oriented assessment, and biomedical and psychosocial treatments for all mental disorders. These methods provided the basis for preparing patients for community living, while preparing staff for major changes in their clinical practice. This paper outlines the process and provides a survey of the results achieved in a quasi-experimental study with 5-year follow-up.

## Method

### Overview (Figure 1)

**Figure 1:**
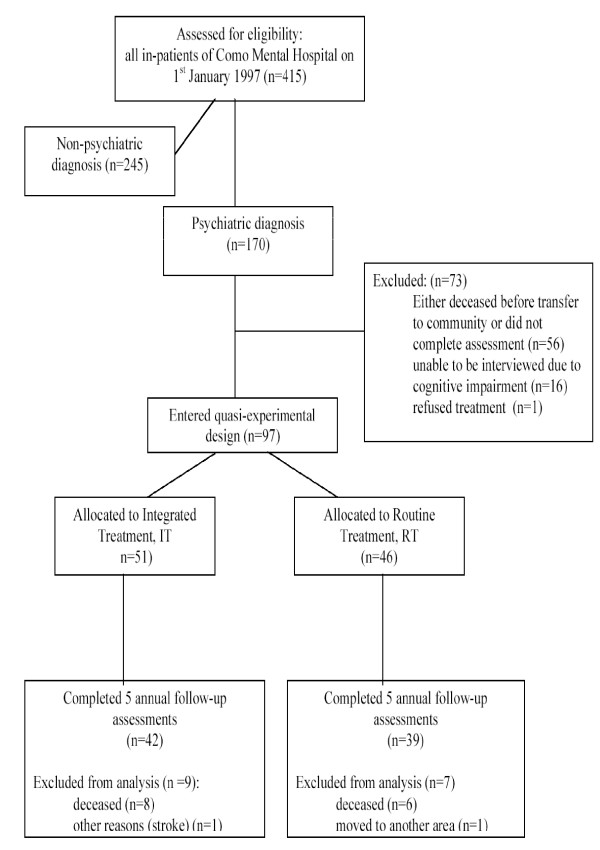


On the 1^st ^January 1997 415 patients were residents of Como Mental Hospital. 170 had a diagnosis of a primary mental disorder that was confirmed by a standardised review of current and past symptoms. In the two years prior to the hospital closure 73 of these cases had died, or were unable or unwilling to be interviewed. Of the remaining 97, 51 were assigned consecutively to an experimental rehabilitation program. It had been intended that all patients would complete this programme prior to discharge, but when the law dictated that the hospital close earlier than expected 46 cases had not yet begun the programme. No selection criteria were used to determine the order in which cases began the rehabilitation earlier. Thus, the two cohorts of patients could be compared in a naturalistic follow-up: 1) those receiving the hospital-based rehabilitation programme, and 2) those who were still awaiting the programme when the hospital closed. Patients in both groups were followed up every year after discharge to assess their clinical and social status.

It was hypothesised that this population suffered from disorders for which a programme of integrated evidence-based biomedical and psychosocial treatments would lead to stable reductions of clinical and social morbidity, with associated increased capacity to achieve personal goals such as independent living and the pursuit of satisfying occupational and social activities. To this end the professional staff began an intensive programme of training in evidence-based assessment and treatment strategies.

### Staff characteristics

Key workers included nurses, social workers and occupational therapists, few of whom had previous specialized mental health training. They were supported by 7 psychiatrists and 1 clinical psychologist. As the process of discharging patients went on there was a parallel movement toward relocating staff to community settings.

### Rehabilitation programme, IT

In 1997 a training project was established for all staff who were caring for mentally disordered residents. This training was part of the international Optimal Treatment Project [10]. The training was adapted to the needs of long-term disabled patients and included workshops on comprehensive standardized biomedical and psychosocial assessments, clarifying patients' personal goals, educating patients about their mental disorders and treatments, optimal pharmacotherapy, early warning signs of exacerbations, assertive community treatment and crisis management, enhancing interpersonal communication and social skills, enhancing personal self-care, structured problem solving and other cognitive behavioural strategies to aid coping with targeted residual psychotic, negative, anxiety and mood symptoms, as well as problems of substance abuse, anger and frustration. This intervention was termed Integrated Treatment, IT [see [10]].

The Italian versions of OTP professional manuals and patient guidebooks were the basis for the professional and patient training sessions [11]. These were educational lesson guides designed to be easily followed by professionals and patients alike. They were based on the principles of error-free learning and practical skills training targeted to the explicit personal goals and key problems of each patient. After practice and discussion in individual and group sessions patients applied the strategies in their actual life situations and reported their outcomes at the next training session, where they received praise and encouragement for their efforts and further coaching to help achieve their goals to the level that they considered satisfactory, before moving on to another goal that they considered likely to improve their current life quality. A total of 100 hours of workshop training and supervision was provided over two-years.

They lived together in groups of 8–10, either in "apartments" or in other residential facilities within confines of the hospital, without restrictions, except for the need to follow straightforward cohabitation rules that were agreed among fellow residents. They were able to practice their skills and work on their goals together with daily staff coaching. Treatment was completed at discharge from the hospital. Although efforts were made to continue this treatment once patients were resident in the community, often this was not feasible, either because staff in the community residences and mental health services were not trained in the methods, or more commonly because managers favoured other approaches.

### Routine Treatment, RT

Patients awaiting IT were treated by the same group of professionals. Pharmacotherapy, nursing care and occupational therapy was provided within a supportive problem-oriented framework. However, no structured psychosocial assessment or treatment protocols were provided.

### Assessment

All patients were assessed by an independent assessor, who was blind to treatment allocation, at the start of the project and at yearly intervals thereafter. The assessor was trained to administer the following ratings to an intra-class reliability of at least 0.80.

#### Clinical

The BPRS-24 [12] was used to assess the severity of psychiatric symptoms.

#### Psychosocial Functioning

The Italian version of Global Assessment of Functioning, GAF, [13] was used to assess psychosocial functioning.

### Data Analysis

In addition to descriptive data, repeated measures analysis of variance using SPSS-PC 10.1.0 was conducted to evaluate trends over time on the entire cohort and interactions between the two treatment groups. In order to compensate for any non-random differences between the two groups the duration of illness, age, gender as well as initial ratings on each specific rating scale were included as covariates. An alpha of .05 was used to define statistical significance.

## Results

### Sample of residents who entered the project (Table 1)

**Table 1:** Characteristics of IT and Routine Treatment patients at the beginning of the hospital closure project

	IT group	RT group
N =	51	46
Age (mean years and range)	57 (35–70)	58 (40–73)
Gender:		
Male (%)	21 (41%)	33 (72%)
Female(%)	30 (59%)	13 (18%)
Duration of Illness (mean years and range)	23 (6–41)	27 (8–40)
Duration of this hospital admission (mean years and range)	16 (6–29)	19 (7–40)
Diagnosis: DSM-IV:		
Schizophrenic Disorders (%)	38 (74%)	37 (81%)
Affective Disorders (%)	7 (14%)	2 (4%)
Anxiety and Personality Disorders (%)	1 (2%)	2 (4%)
Substance Abuse (%)	1 (2%)	5 (11%)
Mental retardation (%)	4 (8%)	-
Died during follow-up period	8 (16%)	6 (13%)
5^th ^year evaluation not available	1 (stroke)	1 (missed interview)
Complete assessments throughout 5 years	42 (82%)	39 (85%)

The characteristics of the sample are documented in Table 1. The mean age was 58 years and was similar in both groups. There was an excess of men in the overall sample (54 vs 43). The proportion of men to women was significantly greater in the routine treatment, RT than in IT. 14 patients died after discharge (8 in IT and 6 in RT). All deaths were from natural causes. Two other cases were unable to complete the 5 annual assessments; 1 IT case had a stroke, and 1 RT case was admitted to forensic hospital in another area.

More than two-thirds had a DSM-IV diagnosis of a schizophrenic disorder. These were equally distributed between the treatment groups. The mean duration of mental disorders was 25 years (range 6 to 41 years). Although the duration of cases entering IT was significantly lower, the respective mean durations of 23 and 27 years would appear to have limited clinical significance. More than 80% had been mentally ill for more than 20 years, and half of these had been resident in the hospital continuously for the past 20 years.

Thus, apart from the disparity of the gender mix, the two cohorts appeared closely matched.

### Residence

Four patients (4.1%) returned to their own homes or to live with family or friends; 89 (92%) went to live in 13 specialized residences for psychiatric patients. Almost all patients were able to choose their own place of residence and most were happy with the choice. However, probably owing to the precipitous hospital closure, there were many subsequent changes, with one quarter changing residence at least once in the 5 years after discharge.

### Homelessness

One RT patient with a history of vagrancy became homeless after repeatedly refusing housing. According to his wishes he was given social care and shelter, including admissions to the general hospital psychiatric ward when he requested it. He continued to receive regular treatment most of the time, but missed most assessment interviews.

### Criminality and Behavioural Problems in the Community

The same homeless patient was arrested twice. Once for threatening behaviour and carrying a weapon (a knife), for which he was not charged. One the second occasion he was charged with burglary and admitted to a forensic hospital. No other problems were reported to police, community services or local authorities.

### Clinical Outcome in the quasi-experimental study (Table 2)

**Table 2:** Clinical and Social Outcome of 81 cases who completed all six baseline and follow-up assessments

			BPRS total		GAF	
Assessment time (months)	Treatment group	N	Mean	Std. Deviation	Mean	Std. Deviation
0	otp	42	37.02	11.46	45.14	10.92
	rt	39	42.05	17.25	37.85	11.68
	total	81	39.44	14.66	41.63	11.80
12	otp	42	36.43	14.15	50.69	13.83
	rt	39	42.44	18.29	41.26	13.71
	total	81	39.32	16.45	46.15	14.48
24	otp	42	40.17	13.72	49.24	13.24
	rt	39	48.00	20.27	39.69	13.70
	total	81	43.94	17.52	44.64	14.22
36	otp	42	40.02	12.00	52.26	12.64
	rt	39	48.64	19.18	41.54	12.61
	total	81	44.17	16.35	47.10	13.66
48	otp	42	35.79	11.15	53.40	12.51
	rt	39	45.59	18.41	40.72	13.10
	total	81	40.51	15.78	47.30	14.23
60	otp	42	37.62	12.77	53.90	14.63
	rt	39	46.33	18.54	40.77	13.69
	total	81	41.81	16.31	47.58	15.57

Eighty-one patients (82%) completed all 6 BPRS interviews; 42 in the IT group and 39 in RT.

On the sum of BPRS items the total cohort of discharged patients showed significant deterioration over the 5-year follow-up period (repeated measures ANOVA with Greenhouse-Geisser correction for lack of sphericity: F = 4.22; df 3.34, 271.74, p = .004). The IT group improved after the program in hospital, then gradually deteriorated in the second and third years in the community before regaining their baseline level. By contrast, the RT group showed significant deterioration from baseline during years 2 and 3 and improved somewhat during years 4 and 5, but did not regain their baseline level.

Repeated measures analysis of variance showed a significant group × time interaction (F = 6.764; df 1,79; p = .011). This significant interaction remained when baseline BPRS, age, sex and duration of illness were entered as covariates (F = 4.802; df 1,75; p = .032). This supports the observation that the IT group remained more stable over the follow-up period whereas a deteriorating trend was observed for RT.

Eighty-two cases completed all GAF assessments. The entire cohort showed a modest but significant trend to improve over the follow-up period (repeated measures ANOVA with Greenhouse-Geisser correction for lack of sphericity: F = 10.04; df 3.622, 293.36; p = .001). Both groups followed this trend and showed significant improvements with time. Most improvement occurred during the first year after discharge. IT cases improved most during the training programme and showed smaller improvements after discharge. RT cases were not assessed at discharge, but showed significant improvements in functioning during the first year of community living, but these were less well sustained.

Repeated measures analysis of variance showed a significant group × time interaction (F = 15.99; df 1,79; p < .001), but on this occasion it just failed to remain significant when the baseline GAF assessment, age, sex and duration of illness were all entered as covariates (F = 3.95; df 1,75; p = .051).

In order to clarify the clinical significance of these findings we constructed an "index of recovery". Cases considered to have made a good recovery were those who had a BPRS total score of between 24 (the minimum) and 30; and in addition had a GAF score of at least 60. At the baseline assessment 8.5% of cases allocated to the IT interventions and 4.8% of those receiving RT were had achieved a good recovery. At 5 years 33.3% of the IT and 10.3% of RT cases met the good recovery threshold. This advantage for IT was significant (Fisher's exact test: p = .016, two-sided).

In order to confirm that the main factor associated with the better clinical and social outcome for the IT cohort was the programme of evidence-based interventions received before discharge, we conducted an ordinal multiple regression analysis with the index of recovery as the dependent variable and the following variables entered into the equation: treatment group, diagnosis (schizophrenia vs. other), age (older or younger than the median age of 62 years), gender, and duration of illness (greater or less than 29 years median). The only variable that was statistically significant in the regression equation was the treatment group allocation. This added support to the hypothesis that the pre-discharge period of intensive rehabilitation contributed to the benefits of those allocated to IT.

## Discussion

The Como Project enabled a large mental hospital in North Italy that provided care for equal proportions of neurologically and psychiatrically disabled patients to be closed over a 3-year period with minimal difficulty. A five-year follow-up showed that most psychiatric patients were resettled in residences in the community that appeared to provide similar social and medical support to that which they had received in the long-stay wards. This could be considered a success, as there was little evidence of serious problems either during or after resettlement, including a low rate of criminal or antisocial activity, death and suicide rates that remained stable and lower than those found in the closure of the mental hospitals in London [14].

While providing adequate humane nursing home facilities for elderly demented patients, and adults with developmental disorders may be considered a success, the same results cannot be hailed as a major achievement for rehabilitation. In the past few decades substantial progress has been made in the biomedical and psychosocial treatment of mental disorders, with associated increased rates of recovery, even for cases who do not receive such treatment until relatively late in the course of their disorders [15]. Although the entire cohort of cases with psychiatric disorders did not show any notable clinical or social deterioration over the 5-year period of assessment at best the trend was for clinical and social stability rather than recovery. However, for the cases that received integrated evidence-based treatment for 6 months prior to discharge, statistical and clinically significant reductions in morbidity were evident. Some gains were lost when this treatment did not continue after hospital discharge. However, after 5 years the benefits of this relatively brief intervention were still clearly evident, and one-third had achieved a good recovery from clinical and social morbidity. In contrast only 10% of those in the comparison group had achieved such a status. These latter results are consistent with the findings of other hospital closure programmes that had not introduced specific evidence-based rehabilitation strategies [16,17].

It is clear that recovery from the symptoms and associated disability of mental illness is a slow process that demands continuous optimal treatment for many years. Residential alternatives to long-stay hospital wards may prove less expensive and reduce the alienation of the severely mentally from community resources and opportunities. But unless they are associated with an improvement in the quality of treatment that is provided, many will remain mere asylums in the community that may lead to increased stigma for such disabled people and calls to re-open the large institutions [18].

Extreme care must be taken in the interpretation of these results, because this naturalistic project had many limitations. Although there was no overt bias in the selection of cases for the intensive rehabilitation programme, the sampling was convenience-based and not random. Matching was good, but not perfect, with traditional prognostic factors favouring the IT cohort. Statistical corrections of baseline differences between the two groups did not change the results substantially. However, replications with more rigorous methodology are essential before it can be concluded that the psychosocial interventions used in this project are efficacious in this group of long-term patients both in facilitating relocation to community care, and in enhancing clinical and social recovery. One such project that uses an identical rehabilitation approach is in progress in Koriyama, Japan, with preliminary results suggesting similar benefits [19]. To date hospital closure programmes have been considered successful if patients have managed to merely relocate to community housing without excessive clinical exacerbations, excessive readmissions to acute or long-term hospital facilities, or involvement with the criminal justice system [2,5,17]. This report is one of the few that has reported positive outcomes from such a project. It is evident that mere re-housing achieves limited benefits, and may even be associated with some deterioration in many cases. However, when this major upheaval in the lives of vulnerable people is accompanied by an effort to provide state-of-the-art biomedical and psychosocial treatment such programmes may contribute to significant long-term clinical and social benefits [9,20,21].

An additional major positive effect has been the relative ease of transition to work in community services of the professional staff that participated in the project. The treatment strategies they learned and applied with limited success to the complicated cases in the hospital were equally useful for cases attending community-based services. The greater success encountered in these settings created considerable enthusiasm for the new work environment. All too often efforts to establish effective community-based services prove difficult when staff lack competence in evidence-based treatment strategies for patients they are expected to treat. The Como Project appeared to circumvent this problem through the training and experiences provided during the hospital closure process. In the same manner that the patients need long-term efficacious treatment, staff need continued supervision and upgrading of their therapeutic competence [22]. This is a problem that is now been tackled in the community-based services that have replaced the Como Mental Hospital.

## Authors' contributions

**AM **designed the project, administered all procedures, analysed and intepreted the data and prepared the manuscript.

**CB **administered the project throughout, managed the database, analysed the data and assisted in preparation of the manuscript.

**EP **organised the staff training, administered the clinical procedures, assisted in the preparation of the manuscript.

**FG **assisted in the staff training, administered the clincial procedures, translated work materials and guidebooks

**EL **prepared the database, assisted in the data analysis

**IRHF **conducted the training and supervision, analysed the data, assisted in preparing the manuscript

## References

[B1] de Girolamo G Cozza M The Italian psychiatric reform. a 20-year perspective Int J Law Psychiatry 2000 23 197 214 10.1016/S0160-2527(00)00030-3 10981267

[B2] Leff J Care in the community. Illusion or reality? 1997 Chichester, John Wiley & Sons

[B3] Lamb R Lessons learned from deinstitutionalization in the US Br J Psychiatry 1993 162 587 592 8149108 10.1192/bjp.162.5.587

[B4] Mollica RF From asylum to community. The threatened disintegration of public psychiatry New Eng J Med 1983 308 367 373 6823241 10.1056/NEJM198302173080705

[B5] Gudeman JE Shore MF Beyond deinstitutionalization. A new class of facilities for the mentally ill New Eng J Med 1984 311 832 836 6472385 10.1056/NEJM198409273111306

[B6] Braun P Kochansky G Shapiro R Greenberg S Gudeman JE Johnson S Shore MF Overview: Deinstitutionalization of psychiatric patients. A critical review of outcome studies Am J Psychiatry 1981 138 736 749 7018273 10.1176/ajp.138.6.736

[B7] Hobbs C Tennant C Rosen A Newton L Lapsley HM Tribe K Brown JE Deinstitutionalization for long-term mental illness: a 6-year evaluation Aust N Z J Psychiatry 2002 36 60 66 10.1046/j.1440-1614.2002.00984.x 11929439

[B8] Kaiser W Hoffmann K Isermann M Priebe S Long-term patients in supported housing after deinstitutionalisation – part V of the Berlin Deinstitutionalisation Study Psychiatric Praxis 2001 28 235 243 10.1055/s-2001-15577 11479831

[B9] Paul GC Lentz RJ Psychosocial Treatment of Chronic Mental Patients 1977 Cambridge MA: Harvard University Press

[B10] Falloon IRH OTP Collaborators Optimal treatment for psychosis in an international multisite demonstration project Psychiatr Serv 1999 50 615 618 10332895 10.1176/ps.50.5.615

[B11] Falloon IRH OTP Collaborators Trattamento Integrato per la Salute Mentale 2000 Salerno: Ecomind Publications

[B12] Ventura J Green MF Shaner A Liberman RP Training and quality assurance with the Brief Psychiatric Rating Scale: 'The drift busters" Int J Methods Psychiatr Res 1993 3 221 244

[B13] American Psychiatric Association Diagnostic and Statistical Manual of Mental Disorders 1993 4 APA: Washington DC

[B14] Trieman N Leff J Glover G Outcome of long stay psychiatric patients resettled in the community: prospective cohort study BMJ 1999 319 13 16 10390451 10.1136/bmj.319.7201.13 PMC28146

[B15] Falloon IRH Montero I Sungur M Mastroeni A Malm U Implementation of evidence-based treatment for schizophrenic disorders: two-year outcome of an international field trial of optimal treatment World Psychiatry 2004 3 104 109 PMC1414683 16633471

[B16] Leff J Trieman N Long stay patients discharged from psychiatric hospitals Br J Psychiatry 2000 176 217 223 10.1192/bjp.176.3.217 10755067

[B17] Barbato A D'Avanzo B Rocca G Amatulli A Lampugnani D A study of long-stay patients resettled in the community after closure of a psychiatric hospital in Italy Psychiatr Serv 2004 55 67 70 10.1176/appi.ps.55.1.67 14699203

[B18] de Girolamo G Picardi A Micciolo R Falloon I Fioritti A Morosini P PROGRES Group Residential care in Italy. National survey of non-hospital facilities Br J Psychiatry 2002 181 220 225 10.1192/bjp.181.3.220 12204926

[B19] Mizuno M Sakuma K Ryu Y Takebayashi T Murakami M Falloon IRH Kashima H The Sasagawa Project: rationale, methods, and recruitment Keio Med J 2005 54 95 101 10.2302/kjm.54.95 16077259

[B20] Thornicroft G Bebbington P Deinstitutionalisation – from hospital closure to service development Br J Psychiatry 1989 155 739 753 2695205 10.1192/bjp.155.6.739

[B21] Economou M Palli A Falloon IRH Violence, misconduct and schizophrenia: Outcome after 4 years of optimal treatment Clinical Practice and Epidemiology in Mental Health 2005 1 3 10.1186/1745-0179-1-3 PMC1151595 15967054

[B22] Senn V Kendal R Trieman N The TAPS project 38: level of training and its availability to carers within group homes in a London district Soc Psychiatry Psychiatr Epidemiol 1997 32 317 322 10.1007/BF00805435 9299924

